# Human Activity Recognition, Monitoring, and Analysis Facilitated by Novel and Widespread Applications of Sensors

**DOI:** 10.3390/s24165250

**Published:** 2024-08-14

**Authors:** Hui Liu, Hugo Gamboa, Tanja Schultz

**Affiliations:** 1 Cognitive Systems Lab, University of Bremen, 28359 Bremen, Germany; tanja.schultz@uni-bremen.de; 2 Laboratory for Instrumentation, Biomedical Engineering and Radiation Physics (LIBPhys-UNL), Faculty of Sciences and Technology, NOVA University of Lisbon, 2820-001 Caparica, Portugal; h.gamboa@fct.unl.pt

## 1. Introduction

The Special Issue *Sensors for Human Activity Recognition* has received a total of 30 submissions so far, and from these, this new edition will publish 10 academic articles.

Scientists in the fields of sensor applications and human activity recognition (HAR) have once again collaborated for this shared academic endeavor. After the first edition of this Special Issue [1] received significant attention, at the request of numerous authors and readers, a second series was launched, for which we received submissions and chose many excellent manuscripts based on a rigorous review, revision, and selection process. This edition brings together the hard work of 50 authors from 14 countries across three continents: Oceania (Australia), Europe (France, Greece, Italy, Australia, Germany, Portugal, Slovenia, Sweden, and the UK), and Asia (China, Pakistan, Saudi Arabia, and Thailand). Of the 51 authors who participated in the first edition [2], less than ten have once again presented their valuable research achievements; the remaining authors are first-time participants.

From hardware to software, from pipelines to applications, from handcrafted features to domain generalization, and from shallow learning to deep and even large language models, the collected literature will provide readers in related fields with state-of-the-art approaches to many challenges in HAR.

## 2. Overview of the Contributions

In the first edition, the literature was introduced in the order specified in the up-to-date HAR research pipeline proposed in [3]. However, such an approach is not adopted in this new edition because, on the one hand, most of the articles selected for this edition involve the vast majority of the links in the pipeline, and some of them even followed the overall HAR research pipeline to formulate their research workflow and plan; on the other hand, the breadth and novelty of the articles’ research fields mean that each have a thoroughly different perspective and reference significance. They will be introduced in alphabetical order of the first letters of the titles; therefore, the order in which the articles appear in this section is not based on their topic.

The subsection titles may not be the most comprehensive summary of the articles’ contributions, but they attempt to highlight their most prominent features and information that sets them apart from other research.

### 2.1. Video-Based Human Activity Recognition (HAR) Using Graph Neural Networks

Research on the use of video as an external sensing method for HAR is increasing and facilitates multiple daily application scenarios. Su and Chen, from Sweden, innovatively used graph neural networks (GNNs) as their model, incorporating semantic content within relational data rather than directly relying on high-dimensional data. They effectively improved the recognition accuracy of activities of daily living (ADLs).

### 2.2. Wearable-Based HAR Plus Localization Using Inertial Plus GPS Data

As carriers of wearable sensors, mobile phones are receiving increasing attention in HAR research [4,5]. As the real-time characteristics of wearable HAR systems face more requirements and challenges [6], smartphones are bound to attract more study. However, the vast majority of this research is based on unimodality, and in particular, accelerometers. Research on superposing localization on HAR is on the rise. First, localization provides not only an auxiliary reference for HAR but also additional motion information for the ending system as well as the HAR results. The authors of this article incorporated GPS sensors into the study of inertial data, expanding mobile phone-based HAR on the above two points: (1) multimodal sensing; (2) superposing localization.

### 2.3. Human Body Movement Characteristics for Virtual Reality-Based Acrophobia Study

Virtual reality should be a potentially safe means of studying and treating acrophobia, as it effectively avoids the environmental risks of physical scenarios of simulated acrophobia in the real world. The Chinese authors set up a series of motion tasks in a virtual reality high-altitude scene to investigate human body movement characteristics. The experimenters established a high-precision classification model, which confirmed that there are differences in the movement patterns of people with and without acrophobia in a high-altitude environment, facilitating quick screening of patients. In addition, a quantitative analysis was presented to provide targeted training guidance to aviation personnel in the future.

### 2.4. Human Activity Counting Using Deep Learning (DL) Maintaining Duration Flexibility

Activity counting is not widely studied but is a potentially useful area of research, similar to the task of event-based automatic segmentation in HAR. In general, data mining methods can be used to count activities in a black-box manner, such as feature-based information retrieval using self-similarity matrices to discover change points and time-series subsequence search to mark pattern reproducibility. Considering event duration flexibility, dynamic time warping and its advanced variants can also be used to count activities through subsequent queries. In this study, the author transformed the task of activity counting from pure statistical analysis to deep learning and achieved robust counting performance on weakly calibrated IMU data for hand-performed activities while maintaining the flexibility of event duration.

### 2.5. Warship Commander Activities for Multisensory Mental Analysis

The authors of this study stated that human performance varies depending on the psychological resources required to successfully complete tasks. In order to monitor users’ cognitive resources in natural scenes, it is necessary not only to measure the needs caused by the task itself, but also to consider the contextual and environmental impacts. A multisource perception dataset of 18 participants was collected based on the warship commander task. The sensor modalities involved include functional near-infrared spectroscopy (fNIRS), electroencephalography (EEG), electrocardiography (ECG), temperature, respiration, and eye tracking, which are used to decode mental effort. The experimental investigation utilized multimodal machine learning approaches that include feature engineering, model optimization, and model selection steps, achieving a reliable classification of mental effort states.

### 2.6. Complex HAR in the Context of Urban Environmental Exposure Research

This work links HAR with the important research field of participatory exposure research, which transforms it into a novel and interesting interdisciplinary task. This type of research usually uses an activity diary, while the authors innovatively used multiple wearable and environmental sensors to perform complex activity recognition in urban stressor exposure studies. At the same time, parameters such as particle concentration, temperature, and humidity were also measured. The recognition experiments were conducted on three shallow learning models. Because the innovation in this research lies in the application scenarios rather than the sensor types, sensor positions, data processing, and machine learning algorithms, the experimental results are not as impressive for less complex activities, and even worse for fuzzy activities such as resting and playing. However, for more complex activities with clear definitions, such as smoking and cooking, strong precision was demonstrated, and these activities are actually essential for the research context.

### 2.7. Accelerometer-Based HAR Using Domain Generalization with Regularization Methods

The concept of domain generalization has been on the research agenda in many fields of artificial intelligence, including HAR. Scientists are trying to find universal means to smoothly connect excellent HAR models to diverse dataset types, including different sensor types, wearable schemes, and data modalities, among others. For example, Motion Units attempt to provide a generalizable, interpretable, and expandable methodology from a human activity modeling perspective by finely distinguishing the stages of daily and sports activities in order to efficiently model the same and different activities in multiple datasets, respectively.

In the article included in this edition, the authors utilized regularization methods to study the domain generalization of HAR and improved the performance of the model. Interestingly, the article confirms again that in the case of sufficient background information, handcrafted features still demonstrate their powerful advantages in HAR [7,8], which is consistent with the conclusions drawn by the authors in the last edition.

It should be supplemented to readers who have conducted in-depth research in this field that recently, studies have shown that designing high-level features based on activity characteristics is a novel approach between traditional handcrafted feature extraction and deep-learning-based automatic feature learning to further improve model interpretability and recognition efficiency [9].

### 2.8. Finger Gesture-Based User Identification Using Radio Frequency Technology

Gesture analysis is also one of the hot topics closely related to HAR [10,11], including finger gesture research. This work applied radio frequency (RF) technology to perceive finger motions to perform the user identification task. The drawback of RF signals is not only their low resolution [12] but also the associated user heterogeneity. To address these challenges, the sensing sensitivity against RF signal interference has been significantly improved through orthogonal signal interference, and subtle individual features have been extracted from less distinct finger motions, such as air-writing digits, through velocity distribution profiling. Under the few-shot model retraining framework based on the first component reverse module, the experimenters efficiently and effectively achieved extensive model robustness and performance in complex environments.

### 2.9. Associating Human Behavior, Manufacture, and Digital Interaction with Fabrication

The integration of coexisting interactive fabrication tools and a dynamic interactive process for fabrication design is proposed in this article. Via virtual–physical integration approaches, designers, manufacturers, and assemblers could be supported in digital fabrication, comprehensively bringing Internet of Things technologies into the co-fabrication space. Human behavior, physical manufacturing, and digital interaction, as the three major components of the system, were focused on, and a seeing–moving–seeing thinking framework was applied to convey the design results.

### 2.10. Facial Expression Understanding Using DL and Multimodal Large Language Models

Facial expressions are subtle changes that occur on the human face. External sensing [13,14,15,16] and physiological signals [17] are increasingly facilitating this area. Similarly to the gesture research introduced in Section 2.8, research on facial expressions is also strongly related to HAR in scientific fields. A notable example is the world-renowned annual FG conference, i.e., the IEEE International Conference on Automatic Face and Gesture Recognition, which clearly prioritizes face, gestures, and body activity computing, analysis, synthesis, and recognition as an equally important direction of research, and which is also implied in their study’s correlations and method cross-referencing.

As the only review included in this edition, this article provides a detailed introduction to the current research status of deep learning and multimodal large language models for facial expression recognition, presenting abundant reference materials for subsequent peer researchers.

## 3. Conclusions and Acknowledgments

Based on the introduction in Section 2, we have extracted ten interesting individual words: graph, localization, acrophobia, counting, mental, exposure, generalization, identification, fabrication, expression. At first glance, none of them seem to be conventionally related to traditional “Sensors for HAR” research, but after careful consideration, they certainly show some subtle connections to HAR which are worth investigating more deeply. The ten articles in this edition present these discoveries in detail to readers without reservation.

In addition to paying tribute to and expressing gratitude for the 50 outstanding contributing scientists, we also want to thank all the diligent and responsible reviewers, as well as MDPI and its editors, who have always supported this Special Issue in the background.

## References

[1] Liu H., Gamboa H., Schultz T. (2023). Sensors for Human Activity Recognition.

[2] Liu H., Gamboa H., Schultz T. (2023). Sensor-Based Human Activity and Behavior Research: Where Advanced Sensing and Recognition Technologies Meet. Sensors.

[3] Liu H., Hartmann Y., Schultz T. A Practical Wearable Sensor-Based Human Activity Recognition Research Pipeline. Proceedings of the 15th International Joint Conference on Biomedical Engineering Systems and Technologies—Volume 5: HEALTHINF.

[4] Kwon Y., Kang K., Bae C. Analysis and evaluation of smartphone-based human activity recognition using a neural network approach. Proceedings of the IJCNN 2015—International Joint Conference on Neural Networks.

[5] Straczkiewicz M., James P., Onnela J.P. (2021). A systematic review of smartphone-based human activity recognition methods for health research. npj Digit. Med..

[6] Hartmann Y., Liu H., Schultz T. Interactive and Interpretable Online Human Activity Recognition. Proceedings of the PERCOM 2022—20th IEEE International Conference on Pervasive Computing and Communications Workshops and other Affiliated Events (PerCom Workshops).

[7] Liu H., Schultz T. ASK: A Framework for Data Acquisition and Activity Recognition. Proceedings of the 11th International Joint Conference on Biomedical Engineering Systems and Technologies—Volume 3: BIOSIGNALS.

[8] Hartmann Y., Liu H., Schultz T. Feature Space Reduction for Multimodal Human Activity Recognition. Proceedings of the 13th International Joint Conference on Biomedical Engineering Systems and Technologies—Volume 4: BIOSIGNALS.

[9] Hartmann Y., Liu H., Schultz T. (2023). High-Level Features for Human Activity Recognition and Modeling. Biomed. Eng. Syst. Technol..

[10] Saini R., Maan V. Human Activity and Gesture Recognition: A Review. Proceedings of the ICONC3 2020—International Conference on Emerging Trends in Communication, Control and Computing.

[11] Mahbub U., Ahad M.A.R. (2022). Advances in human action, activity and gesture recognition. Pattern Recognit. Lett..

[12] Godyak V. (2021). RF discharge diagnostics: Some problems and their resolution. J. Appl. Phys..

[13] Cohen I., Sebe N., Garg A., Lew M.S., Huang T.S. Facial expression recognition from video sequences. Proceedings of the IEEE International Conference on Multimedia and Expo.

[14] Michael P., El Kaliouby R. Real time facial expression recognition in video using support vector machines. Proceedings of the 5th International Conference on Multimodal Interfaces.

[15] Chen J., Chen Z., Chi Z., Fu H. (2018). Facial Expression Recognition in Video with Multiple Feature Fusion. IEEE Trans. Affect. Comput..

[16] Cohen I., Sebe N., Garg A., Chen L.S., Huang T.S. (2003). Facial expression recognition from video sequences: Temporal and static modeling. Comput. Vis. Image Underst..

[17] Veldanda A., Liu H., Koschke R., Schultz T., Küster D. Can Electromyography Alone Reveal Facial Action Units? A Pilot EMG-Based Action Unit Recognition Study with Real-Time Validation. Proceedings of the 17th International Joint Conference on Biomedical Engineering Systems and Technologies—BIODEVICES.

